# Epithelial argininosuccinate synthetase is dispensable for intestinal regeneration and tumorigenesis

**DOI:** 10.1038/s41419-021-04173-x

**Published:** 2021-10-01

**Authors:** Jonathan H. M. van der Meer, Ruben J. de Boer, Bartolomeus J. Meijer, Wouter L. Smit, Jacqueline L. M. Vermeulen, Sander Meisner, Manon van Roest, Pim J. Koelink, Evelien Dekker, Theodorus B. M. Hakvoort, Jan Koster, Lukas J. A. C. Hawinkels, Jarom Heijmans, Eduard A. Struijs, Marja A. Boermeester, Gijs R. van den Brink, Vanesa Muncan

**Affiliations:** 1 Amsterdam UMC, University of Amsterdam, Tytgat Institute for Liver and Intestinal Research, Amsterdam Gastroenterology Endocrinology Metabolism, Meibergdreef 69-71, Amsterdam, The Netherlands; 2 Amsterdam UMC, University of Amsterdam, Department of Gastroenterology and Hepatology, Amsterdam Gastroenterology Endocrinology Metabolism, Meibergdreef 69-71, Amsterdam, The Netherlands; 3 Amsterdam UMC, University of Amsterdam, Department of Oncogenomics, Cancer Center Amsterdam, Meibergdreef 9, Amsterdam, The Netherlands; 4 Department of Gastroenterology and Hepatology, Leiden University Medical Center, Leiden, The Netherlands; 5 Amsterdam UMC, Vrije Universiteit Amsterdam, Department of Clinical Chemistry, Amsterdam Gastroenterology Endocrinology Metabolism, de Boelelaan 1117, Amsterdam, The Netherlands; 6 Amsterdam UMC, University of Amsterdam, Department of Surgery, Meibergdreef 9, Amsterdam, The Netherlands; 7 Roche Innovation Center Basel, F. Hoffmann-La Roche AG, Basel, Switzerland

**Keywords:** Cancer metabolism, Regeneration, Cancer metabolism, Experimental models of disease, Oncogenesis

## Abstract

The epithelial signaling pathways involved in damage and regeneration, and neoplastic transformation are known to be similar. We noted upregulation of argininosuccinate synthetase (ASS1) in hyperproliferative intestinal epithelium. Since ASS1 leads to de novo synthesis of arginine, an important amino acid for the growth of intestinal epithelial cells, its upregulation can contribute to epithelial proliferation necessary to be sustained during oncogenic transformation and regeneration. Here we investigated the function of ASS1 in the gut epithelium during tissue regeneration and tumorigenesis, using intestinal epithelial conditional *Ass1* knockout mice and organoids, and tissue specimens from colorectal cancer patients. We demonstrate that ASS1 is strongly expressed in the regenerating and *Apc*-mutated intestinal epithelium. Furthermore, we observe an arrest in amino acid flux of the urea cycle, which leads to an accumulation of intracellular arginine. However, loss of epithelial *Ass1* does not lead to a reduction in proliferation or increase in apoptosis in vivo, also in mice fed an arginine-free diet. Epithelial loss of *Ass1* seems to be compensated by altered arginine metabolism in other cell types and the liver.

## Introduction

Epithelial tissues in healthy adult organs, such as the intestinal epithelium, are in a state of homeostasis, where the rates of cell division and cell death are in a dynamic equilibrium. Loss of epithelial cells by damage is rapidly compensated for by increased proliferation until the number of differentiated cells is restored and proliferation is reduced to homeostatic levels [1, 2]. This repair process is driven by repopulation of the stem cell pool by surviving stem cells, dedifferentiation of more committed progenitor cells that are able to adopt a stem cell phenotype and hyperproliferation [3, 4]. Multiple parallels can be observed between the process of epithelial repair and oncogenesis, which largely involves the same mechanisms and pathways, with the notable exception that homeostasis is not restored due to the presence of somatic mutations that disrupt the possibility of negative feedback regulation [5, 6].

To provide the cell with its requirements for accelerated growth under challenging conditions, cellular metabolism is grossly altered [7]. For instance, cancer cells often rely on glycolysis instead of oxidative phosphorylation, a phenomenon known as the Warburg effect [8]. In some types of cancer, certain non-essential amino acids may become essential, such as leucine, serine, and arginine [9, 10], while in other types of cancer an increase in the synthesis of amino acids can be observed [11–13]. Because of the similarity between the mechanisms involved in regeneration and oncogenesis, understanding these metabolic changes in cancer could not only provide therapeutic targets for cancer, it may also unveil treatment options for other diseases, like inflammatory bowel disease and several autoimmune disorders, which are characterized by impairment of the regeneration process and intestinal barrier function [14–16].

The amino acid arginine is a direct substrate for protein synthesis, as well as a precursor for nitric oxide, urea, creatine, polyamines, agmatine, proline, and glutamate [17]. Arginine is a conditionally essential amino acid, meaning that availability of arginine becomes critical, mainly during the processes of growth and repair. As mother’s milk contains too little arginine to sustain the rapidly growing embryo and neonate, arginine is synthesized from proline and glutamine that are abundantly present in that milk. The rate-limiting enzyme for this synthesis is argininosuccinate synthetase 1 (ASS1). ASS1 catalyzes a key intermediate step in which citrulline and aspartate are converted to argininosuccinate. Argininosuccinate lyase (ASL) subsequently converts argininosuccinate into arginine and fumarate. As a result, ASS1 is highly expressed by the enterocytes of the small intestine during prenatal and neonatal development. Expression of ASS1 is lost during the suckling-to-weaning transition, when the neonate switches from a diet of milk to solid food [18].

Although the high rate of cellular growth in cancer would suggest that arginine synthesis is essential, the role of arginine biosynthesis in cancer is poorly understood. Several cancer types are characterized by low ASS1 expression, including malignant melanoma, hepatocellular carcinoma, mesothelioma, and prostate cancer [13]. In these cancers, the promoter of the *ASS1* gene is usually epigenetically silenced [11]. Silencing of *ASS1* in these cancers is associated with tumor progression, resulting from increased pyrimidine synthesis, caused by a higher availability of aspartate [19]. Since ASS1-low tumors rely on extracellular arginine for their survival, arginine deprivation therapy, by using arginine-catabolizing enzymes like pegylated arginine deiminase and recombinant arginase, is currently being investigated in several clinical trials [20, 21]. In contrast, other types of cancer, such as colorectal cancer, show an increase in the expression of ASS1 [11, 13, 22].

Recent reports suggest a role for increased ASS1 expression in the growth and survival of colorectal cancer cells [23]. Given the fact that arginine is known to be an important amino acid for tumor growth [24–26], modulation of arginine synthesis could be a therapeutic option to inhibit cancer growth. Additionally, stimulation of arginine synthesis might facilitate epithelial repair in case of a defective intestinal epithelial barrier.

In the context of epithelial repair, it has been shown that *Ass1* whole-body heterozygous mice are more susceptible to irradiation, and particularly suffer from a decreased regenerative capacity of the small intestine [27]. When *Asl*, the enzyme that is the ultimate step in arginine synthesis, is deleted from epithelial cells, in combination with an arginine-free diet, mice treated with dextran sodium sulfate develop more severe intestinal inflammation [28], and silencing of *Asl* inhibits in vitro colorectal cancer cell growth [29].

Although the current literature suggests that ASS1 contributes to intestinal repair and tumorigenesis, its exact function remains unclear. In the present study, we carefully investigated the role of epithelial *Ass1* in intestinal repair and oncogenesis in vitro and in vivo. We utilize murine models where *Ass1* is specifically deleted from intestinal epithelium as well as human colorectal adenomas and cancer tissue.

## Materials and methods

### Patient material

Fresh samples of adenomas and adjacent healthy tissue were collected from patients and snap-frozen immediately after resection in the endoscopy program for the removal of large (>1 cm) colorectal adenomas at the Amsterdam University Medical Centers (location AMC). All patients provided written informed consent (METC2015_206). mRNA was isolated from the tissue samples by mechanical disruption, using the FastPrep-24-5G (MP Biomedicals) in combination with Lysis Tubes S (Qiagen, Cat No./ID: 19091) two times for 1 min, followed by the Allprep DNA/RNA Universal kit (Qiagen, ID 80204).

Paraffin-embedded tissue samples were obtained from the Department of Pathology, Leiden University Medical Center (LUMC, Leiden, the Netherlands), used according to the guidelines of the Medical Ethical Committee of the LUMC, as previously described [30].

### Animals

All animal experiments were performed in accordance with the Animal Ethical Committee guidelines of the Academic Medical Center in Amsterdam, the Netherlands (permit number PVA235/ALC235AC-G). Mice were given ad libitum access to water and standard mouse chow (Teklad 2916, Envigo, Huntingdon, UK) or arginine-free chow (modified C 1069 Amino acids diet, Altromin, Lage, Germany). Mice were housed in a specific pathogen-free barrier environment with 12 h light/dark cycles. Pathogens were tested quarterly in sentinel animals housed in the same room. A tamoxifen-inducible and intestinal epithelium-specific knockout of *Ass1* was obtained, as described previously [31]. For Cre^ERT2^-mediated recombination, mice were injected intraperitoneally with 2 mg of tamoxifen (Sigma, Saint Louis, MO, USA) for 5 consecutive days. At 1 h prior to sacrifice all mice received an intraperitoneal injection of 100 mg/kg BrdU (Sigma-Aldrich, St Louis, MO, USA; 10 mg/ml in PBS). After sacrifice, the intestine was harvested, washed with cold PBS, and sectioned into proximal, middle and distal tissue of the small intestine. For further analysis, tissue was either snap-frozen or formalin-fixed and embedded in paraffin.

**Fig. 1:**
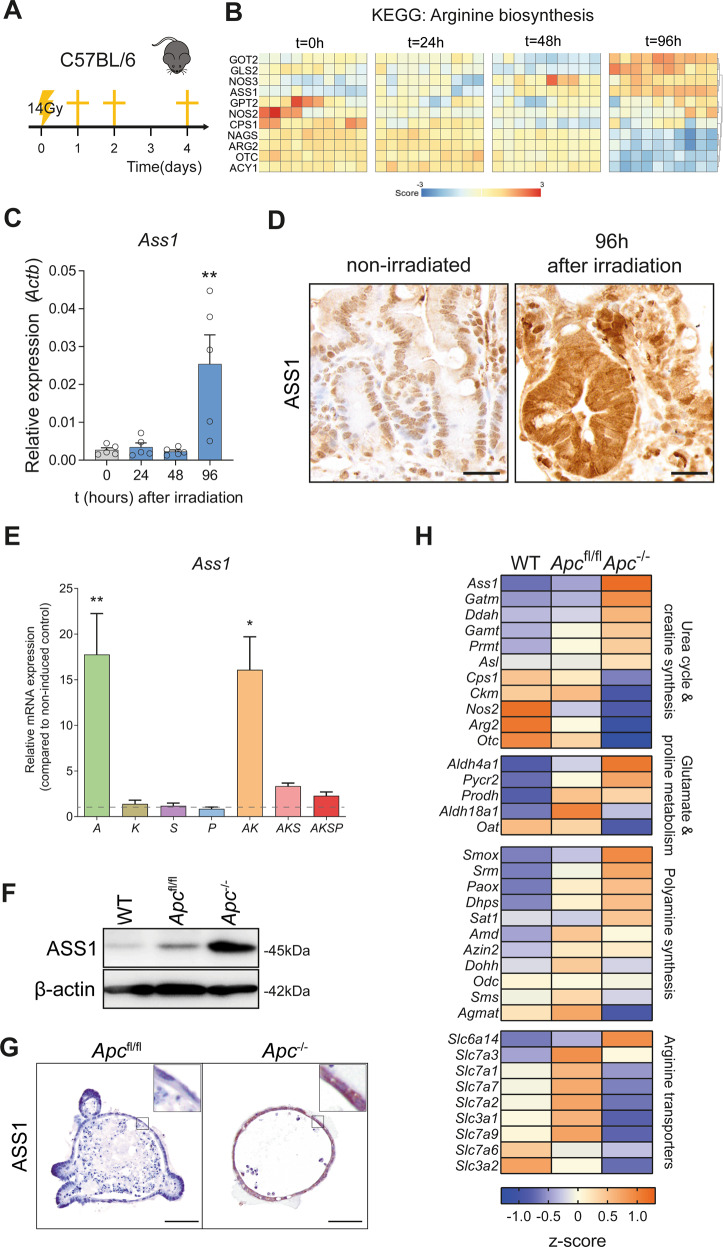
*Ass1* expression increases during regeneration and upon loss of *Apc*. **A** Schematic overview of the mouse model. **B** Heatmap showing the expression of genes in the KEGG geneset ‘Arginine biosynthesis’, from RNA isolated from the intestines of irradiated mice at *t* = 0, 24, 48, and 96 h (*n* = 10 mice per time point). **C** qRT-PCR for *Ass1* on intestinal lysates from the same experiment (*n* = 5 mice per time point), *Actb* is used as a reference gene. **D** Immunohistochemistry for ASS1 in small intestine at *t* = 0 and *t* = 96 h after irradiation. Micrographs show representative images. Scale bar represents 25 µm. **E** qRT-PCR for *Ass1* in murine adenoma-to-carcinoma sequence organoids relative to non-induced control organoid (relative to *Actb*/*Ppia*), A = *Apc*^−/−^, K = *Kras*^G12D/+^, S = *Smad4* shRNA, P = *Trp53*^−/−^ (for single knockout) or *Trp53* shRNA in combination with AKS. ^*^p < 0.05, ^**^p < 0.01 by student’s *t*-test. **F** Western blot for ASS1 and beta-actin in wild type (WT; from C57BL/6 mice), VillinCre^ERT2^ *Apc*^fl/fl^ and VillinCre^ERT2^ *Apc*^−/−^ organoids. **G** Immunohistochemistry for ASS1 in VillinCre^ERT2^ *Apc*^fl/fl^ and VillinCre^ERT2^ *Apc*^−/−^ organoids. Micrographs show representative images. Scale bar represents 50 µm. **H** Heatmap showing qRT-PCR results for various urea cycle and creatine synthesis, glutamate and proline metabolism, polyamine synthesis, and arginine transporter genes.

For the irradiation experiment in Fig. 1, wild-type C57BL/6 mice at 6 weeks of age were treated with 14 Gy whole-body irradiation and euthanized at 24, 48, 72, and 96 h afterwards. For irradiation experiment described in Fig. 4, VillinCre^ERT2^*Ass1*^wt/wt^ and VillinCre^ERT2^*Ass1*^fl/fl^ were first injected with tamoxifen as described above, and 2 weeks later treated with 14 Gy whole-body irradiation. Mice were euthanized 96 h after irradiation.

VillinCre^ERT2^*Ass1*^fl/fl^ mice were crossed to VillinCre^ERT2^*Apc*^fl/fl^ mice in order to generate VillinCre^ERT2^*Ass1*^fl/fl^*Apc*^wt/fl^ and VillinCre^ERT2^*Ass1*^fl/fl^*Apc*^fl/fl^ mice. VillinCre^ERT2^*Ass1*^fl/fl^*Apc*^wt/fl^ heterozygous mice were euthanized 16 weeks after tamoxifen-induced recombination. Adenomas were counted in formalin-fixed tissue through a stereoscopic microscope. VillinCre^ERT2^*Ass1*^fl/fl^*Apc*^fl/fl^ homozygous mice were euthanized 5 days after tamoxifen induction. VillinCre^ERT2^*Ass1*^fl/fl^*Apc*^fl/fl^ mice fed an arginine-free diet were euthanized 4 days after tamoxifen induction.

### Mouse intestinal-crypt isolation and organoid culture

Small intestinal single crypts were isolated from C57BL/6, VillinCre^ERT2^*Apc*^fl/fl^ (two types: exon 15 *Apc*^15lox/15lox^ and exon 14 *Apc*^580S/580S^ separately tested), VillinCre^ERT2^*Apc*^fl/fl^*Kras*^G12D/wt^, VillinCre^ERT2^*Ass1*^fl/fl^, VillinCre^ERT2^*Apc*^fl/fl^ *Ass1*^fl/fl^, and *Trp53*^F2-10/F2-10^ mice, and cultured in Matrigel (BD, Franklin Lakes, NJ, USA). Crypts were harvested by incubating opened small intestines in PBS containing 2 mM EDTA. The epithelium was released by vigorous shaking and crypts were separated by using a 70 μm cell strainer. Single crypts were cultured in EGF, Noggin, R-spondin (ENR) medium, containing Advanced DMEM medium (Invitrogen/Thermo Fisher, Waltham, MA, USA) supplemented with 1% penicillin/streptomycin, 1% HEPES buffer, and 1% GlutaMAX, 50 ng/ml EGF, 20% Noggin conditioned medium, 10% R-spondin conditioned medium, 1X B-27 supplement, 1X N-2 supplement (all from Invitrogen), and 1.25 mM *N*-acetylcysteine (Sigma). The organoids were then passaged weekly by mechanical disruption. Organoids with shRNAs against *Smad4* and *Trp53* were generated as previously described [32, 33]. Cre-mediated recombination in organoids was established by adding 1 μg/ml 4-hydroxytamoxifen to the culture medium for 24 h. Photographs were taken with a Leica DMi8 microscope and analyzed with ImageJ (version 1.52p, http://imagej.nih.gov/ij/).

Single-cell clonogenicity experiments were performed as previously described [33]. In short, Matrigel was dissolved using cell recovery solution for 30 min on ice. Next, organoids were dissociated to single cells after 7 min of 37 °C incubation with TrypLE (Gibco/Thermo Fisher). The absolute number of cells per condition was equalized using a particle counter (Z-series, Beckman Coulter, Fullerton, CA, USA) and 20,000 cells were plated in a 48-well plate. The number of outgrowing organoids was quantified 3–5 days after seeding, and presented as mean number of four wells.

### RNA extraction and qRT-PCR

RNA was extracted from organoids using the Isolate II RNA Mini Kit (Bioline, London, UK) following the manufacturer’s instructions. The intestinal tissue RNA was extracted with TRI Reagent (Sigma-Aldrich). Complementary DNA was synthesized from mRNA using 10 µM Oligo-dT (ThermoFischer Scientific), 5 µg/ml random hexamer primers (Promega, Madison, USA), 1 mM DNTP-mix, 20 U/reaction RiboLock RNase, and 100 U/reaction RevertAid reverse transcriptase (all ThermoFischer Scientific). Quantitative RT-PCR was performed on a CFX96TM Real-Time System (Bio-Rad Laboratories, Hercules, CA, USA) using Sensifast SYBR green (Bioline) according to the manufacturer’s protocol. mRNA levels were normalized against the indicated reference gene and relative gene expression was calculated with the 2^−ΔΔCT^ method [34].

For the microarray, RNA was amplified using a TotalPrep RNA amplification kit for Illumina (Illumina, San Diego, CA, USA) and labeled using a cRNA labeling kit for Illumina Arrays (Illumina, San Diego, CA, USA), followed by hybridization with Illumina Ref8 v2.0 mouse slides, following the manufacturer’s protocol. Expression profiles were deposited in the GEO repository GSE179777. Initial normalization was performed using Genome studio software (v2.0, Illumina, San Diego, CA, USA). Further analysis was done with R2 Bioinformatics platform (http://r2.amc.nl, AMC, Amsterdam, The Netherlands).

### Western blot

Samples were run on 10% SDS-PAGE gels under reducing conditions and then transferred to nitrocellulose membranes (GE Health Care, Zeist, The Netherlands). Membranes were blocked by incubation in 5% BSA in TBST (TBS + 0.1% Tween-20) for 2 h at room temperature (RT) and subsequently incubated with anti-mmASS1 (1:10,000) [35], anti-hsASS1 (1:300, clone C104588, Sigma, Deisenhofen, Germany), or β-actin (1:100,000, clone AB1978, Sigma, Deisenhofen, Germany) antibodies in 2% BSA in TBST overnight at 4 °C. After incubation membranes were washed three times with TBST, incubated with HRP-conjugated secondary antibodies (1:2000, Dako) in 2% BSA in PBST for 2 h at RT. Expression was detected by Lumilight Plus (Roche). When combining proteins of similar kDa, blots were stripped in stripping buffer (ThermoFischer Scientific) for 10 min at RT and incubated with the other antibody of the same kDa as described above.

### HPLC

Blood was collected into heparin-coated tubes and centrifuged at 500*g* for 5 min at 4 °C. Plasma was added to sulfosalicylic acid, vortexed, and stored at −80 °C. Amino acid concentrations were determined by HPLC as previously described [36].

### Stable-isotope incubation and LC-MS analysis

Organoids were incubated for 96 h in ENR medium enriched with stable-isotope-labeled L-citrulline (ureido-^13^C; 3,3,4-D_3_; Buchem, The Netherlands) or stable-isotope-labeled L-aspartic acid (^13^C4; D3; 15 N, CDNLM-6803-PK; Buchem, The Netherlands). Thereafter, culture media and cell pellets were collected and stored at −80 °C. Kinetics were investigated by measuring isotope‐labeled intermediates with liquid chromatography tandem mass spectrometry (LC‐MS/MS) as previously described [37].

### Griess test

Griess reagent containing 2.3 ml phosphoric acid (85%), 1 g sulfanilamide, 0.1 g naphtylethylenediamine (both Sigma, Deisenhofen, Germany), and 97.7 ml water. Standards from 0 to 1000 µM were prepared from sodium nitrite (NaNO_2_; Merck, Darmstadt, Germany). Griess reagent was added in a 1:1 ratio to medium samples and standards. Absorbance was measured at 540 nm in a BioTek Synergy HT microplate reader (BioTek Instruments, Winooski, VT, USA).

### Immunohistochemistry

Paraffin-embedded tissue was sectioned freshly (4.5 μm) and dried overnight at 37 °C. Slides were deparaffinized with xylene and rehydrated in a graded series of ethanol. Endogenous peroxidase activity was blocked with 3% H_2_O_2_ in methanol. For antigen retrieval, tissue was cooked in 0.01 M sodium citrate solution (pH 6.0) for 20 min. Non-specific binding was prevented by incubation with PBT (phosphate-buffered saline, bovine serum albumin 10 mg/ml, and Triton X-100 0.1%). Tissue sections were incubated overnight with a primary antibody.

Mayer’s hematoxylin (Sigma-Aldrich) was used as nuclear counter stain. The following antibodies were used: anti-mmASS1 (1:2000) [35], anti-hsASS1 (1:2000, clone C104588; Sigma, Deisenhofen, Germany), and anti-BrdU (1:500, clone BMC9318; Roche, Woerden, The Netherlands). Images were captured with an Olympus BX51 microscope.

### Statistical analysis

Data are presented as mean and standard error of the mean (SEM). Student’s *t*-test was used to analyze data with two groups. One-way ANOVA with Tukey’s multiple comparison test was performed for data with more than two groups. For statistical analysis, GraphPad Prism (version 7, La Jolla, CA: GraphPad Software Inc.) was used. A *p*-value < 0.05 was considered statistically significant.

## Results

### *Ass1* is highly expressed in repair of the intestinal epithelium and selectively upregulated in *Apc*-mutated organoids

To investigate alterations in pathways associated with amino acid metabolism in the regenerating intestinal epithelium, we exposed mice to 14 Gy whole-body gamma irradiation (Fig. 1A). In this model of damage and repair, the intestinal epithelium initially undergoes extensive cell death, resulting in a phase of epithelial repair 48–96 h after the irradiation with epithelial hyperproliferation and crypt multiplication through a process of elongation and fission. Microarray analysis on intestinal lysates at 96 h after irradiation revealed *Ass1* to be highly significantly upregulated compared to non-irradiated mice (Fig. 1B). This was confirmed by qRT-PCR and immunohistochemistry (Fig. 1C, D). Hyperproliferative crypts showed especially strong cytosolic staining of ASS1 in line with previous observations [27]. The epithelium of non-irradiated wild-type mice expressed little to no ASS1 as expected. Since it has been suggested that ASS1 is similarly highly expressed in colorectal adenomas and carcinomas, we investigated mRNA expression of *Ass1* in murine organoids in which the main driver mutations associated with the adenoma-to-carcinoma sequence were modeled (*Apc*, *Kras*, *Smad4*, and *Trp53*) [38]. *Ass1* expression was markedly upregulated in organoids carrying loss-of-function of tumor suppressor gene *Apc* (Fig. 1E), whereas the other mutations did not show elevated *Ass1* expression. Partial or complete loss of the tumor suppressor genes *Smad4* and *Trp53* tended to lower *Apc-*induced *Ass1* expression (Fig. 1E). Increased expression of ASS1 in *Apc*^−/−^ organoids was confirmed at the protein level (Fig. 1F, G). In conclusion, only the *Apc* mutation upregulated *Ass1* in intestinal organoids, whilst mutations in *Kras*, *Smad4*, and *Trp53* did not. This suggests that upregulation of ASS1 is specifically associated with activation of the Wnt pathway in intestinal epithelial cells.

### Loss of *Apc* affects multiple genes associated with the urea cycle

Since *Ass1* is upregulated after *Apc* loss, we next investigated the effect of loss of *Apc* on pathway components and associated genes of the urea cycle by qRT-PCR. Many genes that are part of the urea cycle, as well as genes associated with glutamate metabolism, proline metabolism, and polyamine synthesis (Supplemental Fig. 1A) were differentially expressed after loss of *Apc* (Fig. 1H). Expression of *Arg2*, *Nos2*, and *Otc*, central components of the urea cycle that are involved in conversion of arginine, was reduced in the context of *Apc* loss (Fig. 1H and Supplemental Fig. 1B). Furthermore, known arginine transporters, which mediate uptake of arginine, were mostly downregulated (Fig. 1H). Together, increased synthesis of arginine, and decreased conversion and decreased uptake of arginine suggest that *Apc*-deleted cells may be reliant on autologous synthesis of arginine.

### *ASS1* expression correlates with *APC* mutations in CRC cell lines and upregulated in human adenomas

**Fig. 2:**
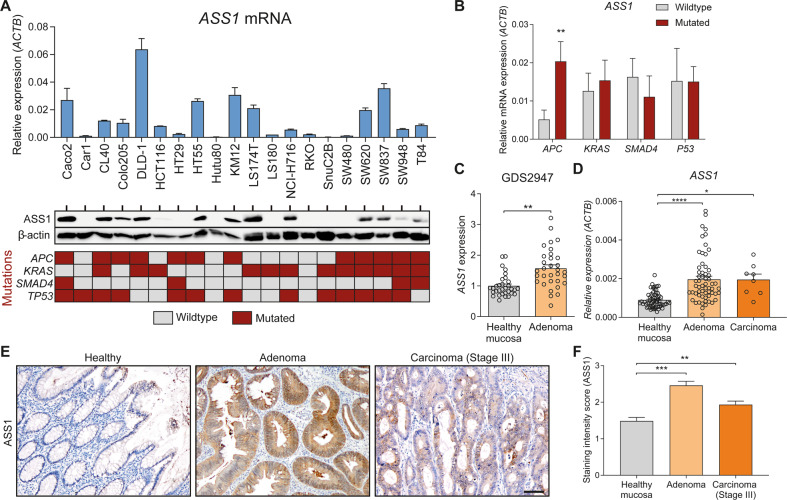
ASS1 expression is upregulated and correlates with *APC* mutations in human colorectal adenomas and carcinomas. **A***ASS1* mRNA and ASS1 protein expression in 20 different colorectal cancer cell lines. Per cell line the mutational status of *APC*, *KRAS*, *SMAD4*, and *TP53* is indicated below. **B** *ASS1* mRNA expression in the same 20 colorectal cancer cell lines as in (**A**), separated by their mutational status. ^**^p < 0.01 by Kruskal-Wallis test. **C** *ASS1* expression in paired samples of normal mucosa and colorectal adenoma tissue (*n* = 32) from the publicly available expression dataset GDS2947. ^****^p < 0.0001 by Wilcoxon signed rank test. **D** qRT-PCR for *ASS1* in tissue from 60 adenoma and 8 carcinoma samples with paired controls. ^*^p < 0.05, ^****^p < 0.0001 by Wilcoxon signed rank test. **E** immunohistochemistry for ASS1 in healthy (*n* = 70), adenoma (*n* = 12), and carcinoma (*n* = 75) tissue. Micrographs show representative images. Scale bar indicates 100 µm. **F** Quantification of (**E**), which was scored in a blinded manner by two independent observers. ^**^p < 0.01, ^***^p < 0.001 by one-way ANOVA with Tukey’s multiple comparisons test.

Next, we measured *ASS1* mRNA and ASS1 protein expression in human colorectal cancer cell lines with a known mutational profile, as performed previously by others [39]. The different cell lines expressed ASS1 to various degrees (Fig. 2A). We related the expression level of *ASS1* to mutations in *APC*, *KRAS*, *SMAD4*, and *TP53* in these cell lines and observed a significant correlation between *ASS1* expression and *APC* mutations, but not for other mutations, microsatellite instability (MSI), or CpG island methylator phenotype (CIMP) status (Fig. 2B and Supplemental Fig. 2A, B), in line with our findings in mouse organoids. In a publicly available gene expression dataset containing healthy and adenomatous tissue from patients (GDS2947 [40]), we observed increased *ASS1* expression (Fig. 2C). In a cohort of RNA samples isolated from 60 adenoma and 8 carcinoma samples paired with healthy control tissues from the same patients (Fig. 2D) and in a separate cohort of paraffin-embedded tissue from 70 healthy, 12 adenoma and 75 stage III carcinoma patients, a similar increase of ASS1 protein expression was observed (Fig. 2E and Supplemental Fig. 2C).

### Loss of *Apc* results in altered arginine metabolism

**Fig. 3:**
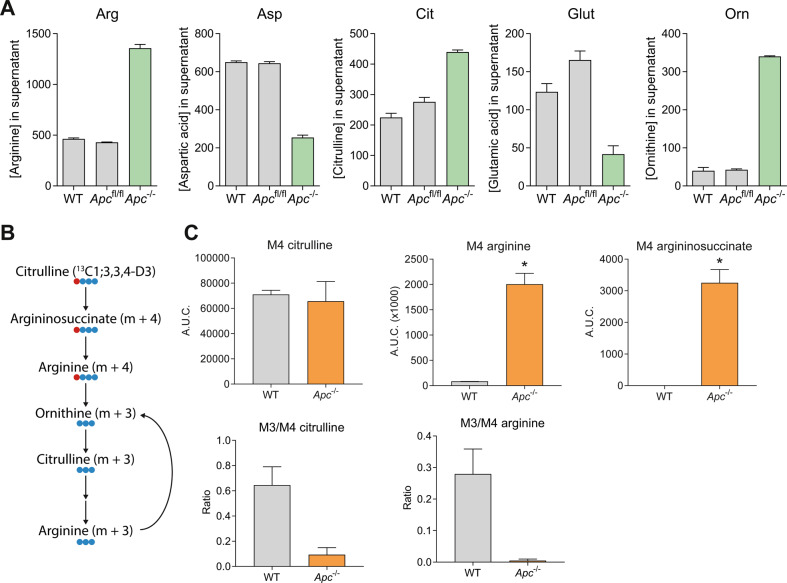
Loss of *Apc* leads to altered amino acid consumption and synthesis. **A** Amino acid concentrations as measured by HPLC in supernatant of wild-type, VillinCre^ERT2^ *Apc*^fl/fl^, and VillinCre^ERT2^ *Apc*^−/−^ organoids. **B** schematic overview of labeling. **C** LC-MS measurement of intracellular M4 citrulline, M4 arginine, and M4 argininosuccinate, in wild-type and VillinCre^ERT2^ *Apc*^−/−^ organoids after incubation with labeled M4 citrulline (13C1;3,3,4-D3), and ratios between M3/M4-labeled citrulline and arginine. ^*^p < 0.05 by student’s *t*-test.

In order to evaluate the effect of the *Apc* mutation in mouse intestinal epithelial organoids on arginine metabolism, we measured amino acid concentrations in the standard ENR medium of wild-type and *Apc*^−/−^ organoids by HPLC. The *Apc*-deleted organoids showed an increased arginine and ornithine concentration, while aspartic acid, glutamic acid, and glutamine were decreased compared to wild-type organoids (Fig. 3A). This shows that the *Apc* mutation results in functional changes in amino acid consumption in intestinal epithelial cells.

Next, we incubated organoids with labeled citrulline (13C1;3,3,4-D3) and used LC-MS to measure the effect of the altered expression of urea-cycle enzymes on the amino acid flux. The labeling in this synthesized citrulline was designed to lose one mass (from M4 to M3) after making a full cycle in the urea cycle (Fig. 3B and Supplemental Fig. 3A). We found increased labeling in argininosuccinate and arginine in *Apc*-mutant organoids, as was expected due to the increased expression of ASS1 (Fig. 3C). We observed a decrease in the ratio of M3/M4 arginine and citrulline in *Apc*-deleted organoids, suggesting inhibition of the flux of the urea cycle. Conversion of arginine into citrulline via ornithine was strongly inhibited, correlating with decreased expression of *Arg2* and *Otc* in *Apc*-mutant versus wild-type organoids. No increase in NO production was seen as measured using the Griess test, which chemically detects nitrite ions (Supplemental Fig. 3D). The combined increase of arginine synthesis through ASS1 and decreased conversion of arginine to ornithine and citrulline would lead to an accumulation of arginine. We hypothesized that the arginine was being used for polyamine synthesis. However, no differential labeling was measured in spermine/spermidine. It has been suggested that knockdown or inhibition of *Ass1* reduces fumarate levels, which leads to inhibition of growth in colorectal cancer cell lines [23]. To test whether the upregulation of *Ass1* in the *Apc*^−/−^ organoids led to an increase in fumarate, we incubated the organoids with labeled aspartic acid (Supplemental Fig. 3B). However, no change in labeling was found in fumarate (Supplemental Fig. 3C).

Therefore, we concluded that upon deletion of *Apc*, citrulline is increasingly converted into arginine. Yet, this arginine is not further converted into other metabolites and the urea cycle does not flux, which ultimately results in the observed accumulation of arginine (Fig. 3A).

### Epithelial *Ass1* is not required for intestinal epithelial regeneration after irradiation

**Fig. 4:**
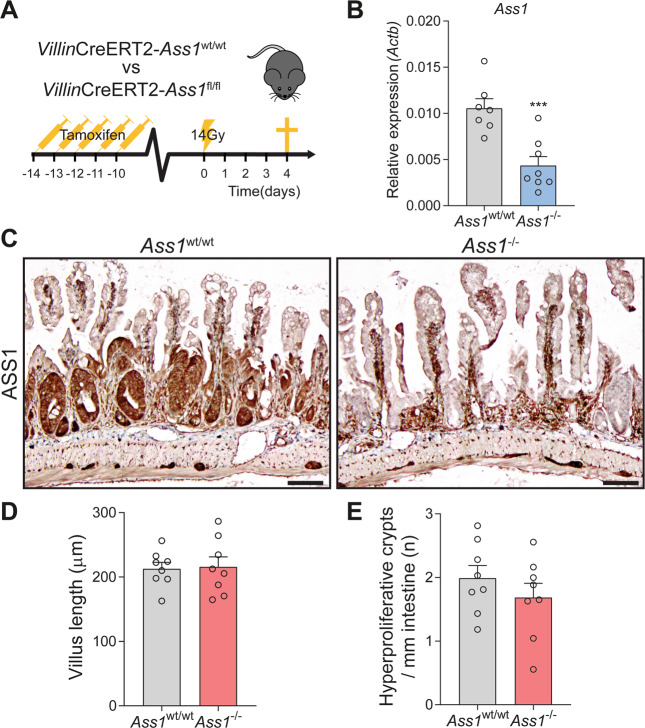
*Ass1* knockout in the intestinal epithelium does not affect regeneration after damage by irradiation. **A** Schematic overview of the mouse model. **B** qRT-PCR for *Ass1* in small intestinal lysates of *Ass1*^wt/wt^ and *Ass1*^−/−^ mice 96 h after irradiation. *Actb* was used as a reference gene. ^***^p < 0.001 by Student’s *t*-test. **C** Immunohistochemistry for ASS1 in small intestinal tissue of *Ass1*^wt/wt^ and *Ass1*^−/−^ mice 96 h after irradiation. Representative images shown. Scale bar indicates 100 µm. **D** Average villus length. **E** Amount of hyperproliferative crypts per mm intestine. *n* = 8 mice per group.

We next set out to investigate the potential usage of excess arginine under in vivo physiological conditions. As mentioned above, whole-body *Ass1* heterozygosity has been shown to impair the regenerative capacity in the small intestine after irradiation [27]. We hypothesized that this effect might be due to epithelial overexpression of *Ass1* and excess arginine production. To test this, we crossed VillinCre^ERT2^ mice, in which Cre is activated after administration of tamoxifen in *Villin*-positive epithelial cells, to *Ass1*^wt/wt^ and *Ass1*^fl/fl^ mice to create mice in which *Ass1* can be specifically deleted from the intestinal epithelium [31]. At 2 weeks after intraperitoneal injection of tamoxifen, the mice were exposed to whole-body 14 Gy of ionizing radiation (Fig. 4A). Epithelial knockout of *Ass1* was efficient as shown by qRT-PCR on tissue lysates and immunohistochemistry (Fig. 4B, C). However, the villus length and the number of regenerating crypts per mm of intestine were not affected by the loss of *Ass1* in the intestinal epithelium (Fig. 4D, E). These data suggest that epithelial upregulation of *Ass1* is not required for intestinal hyperproliferation observed after irradiation as a part of regenerative response.

### Epithelial expression of *Ass1* is not required for intestinal tumorigenesis

**Fig. 5:**
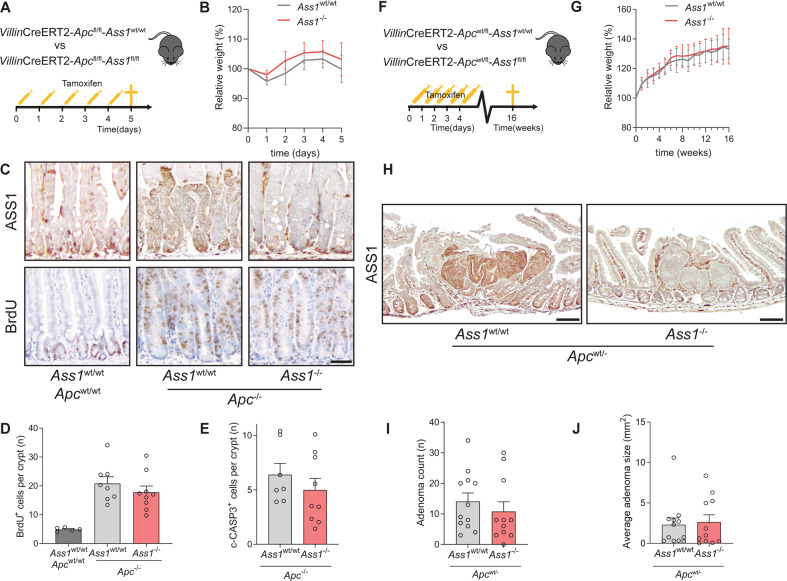
*Ass1* knockout in the intestinal epithelium does not affect tumorigenesis in vivo. **A** Schematic overview of the *Apc*^fl/fl^ mouse model. **B** Relative weight compared to start tamoxifen injections. **C** Immunohistochemistry for ASS1 (upper panels) and BrdU (lower panels) of *Ass1*^wt/wt^ and *Ass1*^−/−^ mice 5 days after recombination. Representative images are shown. Scale bar represents 50 µm. **D** Amount of BrdU-positive cells per crypt in the small intestine. **E** Amount of cleaved caspase-3-positive cells per crypt in the small intestine. **F** Schematic overview of the *Apc*^wt/fl^ mouse model. **G** Relative weight compared to start tamoxifen injections. **H** Immunohistochemistry for ASS1 in adenomas in *Ass1*^wt/wt^ and *Ass1*^−/−^ mice. Scale bar represents 100 µm. **I** Amount of adenomas in both groups. **J** Average adenoma size.

To investigate the effect of loss of *Ass1* in a model of intestinal epithelial adenomatous transformation, we generated VillinCre^ERT2^ *Ass1*^wt/wt^ *Apc*^fl/fl^ and VillinCre^ERT2^ *Ass1*^fl/fl^ *Apc*^fl/fl^ mice (Fig. 5A). In this model, the entire intestinal epithelial layer becomes hyperproliferative within 4 days after tamoxifen-induced recombination of *Apc* [41]. *Ass1* knockout did not have an effect on body weight (Fig. 5B), epithelial cell proliferation (Fig. 5C, D), or apoptosis (Fig. 5E).

To examine the role of epithelial *Ass1* on spontaneous adenoma formation in *Apc* heterozygous epithelium, we crossed these VillinCre^ERT2^ *Ass1*^wt/wt^ and VillinCre^ERT2^ *Ass1*^fl/fl^ to *Apc*^fl/fl^ mice. VillinCre^ERT2^ *Apc*^wt/fl^ heterozygous mice develop adenomas mostly in the small intestine approximately 16 weeks after tamoxifen induction (Fig. 5F). Loss of *Ass1* did not affect body weight (Fig. 5G). In adenomas of wild-type mice, ASS1 protein expression was increased (Fig. 5H; left panel), although not all adenomatous cells expressed high levels of ASS1. *Ass1*^−/−^ mice showed clear loss of ASS1 in all *Villin*-positive cells at 16 weeks after induction (Fig. 5H; right panel). At 16 weeks after recombination, we did not observe any effect of *Ass1* knockout on the amount or size of the adenomas that were formed (Fig. 5I, J). In conclusion, loss of epithelial *Ass1* does not affect epithelial transformation nor tumor progression after acute loss of both *Apc* alleles nor adenomagenesis in *Apc* heterozygous epithelium in vivo. These data suggest that epithelial ASS1 is dispensable for the earliest stages of *Apc*-driven intestinal tumorigenesis.

### *Ass1* only contributes to growth under low-arginine conditions

**Fig. 6:**
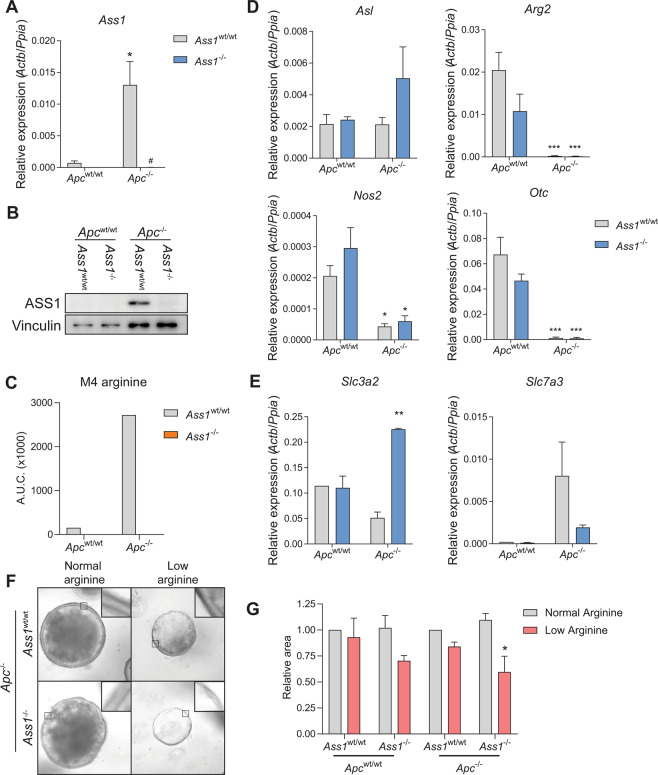
Growth of *Ass1*-deficient small intestinal organoids is impaired in arginine-low conditions only and arginine transporters could compensate for loss of *Ass1*. **A** qRT-PCR for *Ass1* in *Ass1* and *Apc* knockout organoids. *Actb* and *Ppia* are used as reference genes. ^*^p < 0.05 compared to *Ass1*^wt/wt^ *Apc*^wt/wt^ organoids, ^#^*p* < 0.05 compared to *Ass1*^wt/wt^ *Apc*^−/−^ organoids by one-way ANOVA with Tukey’s multiple comparisons test. **B** Western blot for ASS1 and Vinculin in *Ass1* and *Apc* knockout organoids. **C** Intracellular LC-MS measurements of M4 arginine in organoids cultured with M4 citrulline (U-13C;3,3,4-D3). **D** qRT-PCR for indicated urea-cycle genes. **E** qRT-PCR for indicated amino acid transporters. ^*^p < 0.05, ^***^p < 0.001, ^****^p < 0.0001 by one-way ANOVA with Tukey’s multiple comparisons test. **F** Microscopic bright-field images of organoids cultured in regular and low-arginine medium. Representative images are shown. Scale bar represents 100 µm. **G** Quantification of organoid size cultured in regular and low-arginine medium (*n* = 3 independent experiments) ^*^p < 0.05 student’s *t*-test.

Although no visible phenotypic intestinal differences were observed in all the murine models we examined, we further investigated whether loss of ASS1 affects epithelial cells in in vitro organoid cultures. To this end, we isolated intestinal organoids from VillinCre^ERT2^ *Ass1*^wt/wt^, VillinCre^ERT2^ *Ass1*^fl/fl^, VillinCre^ERT2^ *Ass1*^wt/wt^ *Apc*^fl/fl^, and VillinCre^ERT2^ *Ass1*^fl/fl^ *Apc*^fl/fl^ mice. In these purely epithelial cell cultures, efficient knockout of *Ass1* was achieved (Fig. 6A, B) upon tamoxifen induction, confirmed by the inability to synthesize labeled arginine after incubation with labeled citrulline demonstrating the loss of ASS1 function (Fig. 6C). Expression levels of other genes in the urea cycle were unaffected by the loss of *Ass1* (Fig. 6D). Loss of *Ass1* also did not affect expression of Wnt target gene *Axin2* or stem cell markers *Lgr5* and *Ascl2* (Supplemental Fig. 4A), neither did it affect stem cell function as assessed by seeding single cells in a clonogenic assay (Supplemental Fig. 4B). Notably, amino acid transporters *Slc3a2* (encodes for 4F2 cell-surface antigen heavy chain) and *Slc7a3* (encodes for cationic amino acid transporter 3) showed up- and downregulation, respectively (Fig. 6E and Supplemental Fig. 4C). We reasoned that the upregulation of *Slc3a2* could compensate the loss of *Ass1* by allowing increased influx of arginine from the medium into the epithelial cells. In line with our in vivo findings, *Ass1* knockout did not affect organoid size in regular arginine-rich medium (Fig. 6F, G). However, when the organoids were cultured in arginine-low medium, *Ass1*-deficient organoids were significantly smaller in size. Also, the lumen of arginine-deprived organoids seemed to contain less cell debris, suggestive of a lower cell-turnover rate and the cell volume appeared flattened (Fig. 6F).

These data suggest that, in high-arginine conditions *Ass1* does not contribute to organoid growth, possibly due to increased arginine uptake from the medium. However, in low-arginine conditions loss of *Ass1* does affect organoid growth.

### Epithelial *Ass1* does not affect intestinal tumorigenesis in mice fed an arginine-free diet

**Fig. 7:**
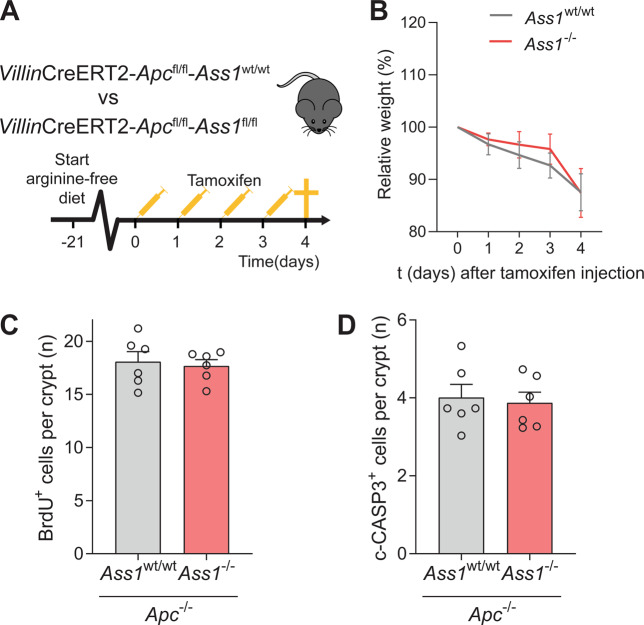
*Ass1* knockout in the intestinal epithelium does not affect *Apc-*mediated hyperproliferation in mice fed an arginine-free diet. **A** Schematic overview of the mouse model. **B** Relative weight compared to start tamoxifen injections. **C** Amount of BrdU-positive cells per crypt in the small intestine. **D** Amount of cleaved caspase-3-positive cells per crypt in the small intestine.

Since loss of epithelial *Ass1* did not inhibit tumorigenesis and hyperproliferation in vivo with arginine present in the diet, and we noted an effect on growth in low-arginine medium in vitro, we repeated the *Apc* homozygous model (Fig. 5) with VillinCre^ERT2^ *Ass1*^wt/wt^ *Apc*^fl/fl^ and VillinCre^ERT2^ *Ass1*^fl/fl^ *Apc*^fl/fl^ mice in arginine-free conditions: Mice were fed arginine-free chow during 3 weeks prior to tamoxifen-induced recombination (Fig. 7A). No difference in weight was observed between the two groups, although the arginine-free diet seemed to lead to increased weight loss overall compared to mice fed regular chow (Figs. 7B and 5B). Although mice were deprived from arginine, the intestinal epithelium in *Ass1*-deficient mice showed the same levels of proliferation as *Ass1* wild-type littermate controls (Fig. 7C). Furthermore, apoptosis was also not affected in VillinCre^ERT2^ *Ass1*^fl/fl^ *Apc*^fl/fl^ mice fed an arginine-free diet (Fig. 7D). This shows that even in arginine-deprived conditions *Ass1* does not play a role in the regenerating and adenomatous intestinal epithelium, excluding the possibility that excess arginine from the diet would obscure a potential role for *Ass1* in our previous experiments.

### Adaptation by the liver potentially compensates for intestinal loss of *Ass1*

**Fig. 8:**
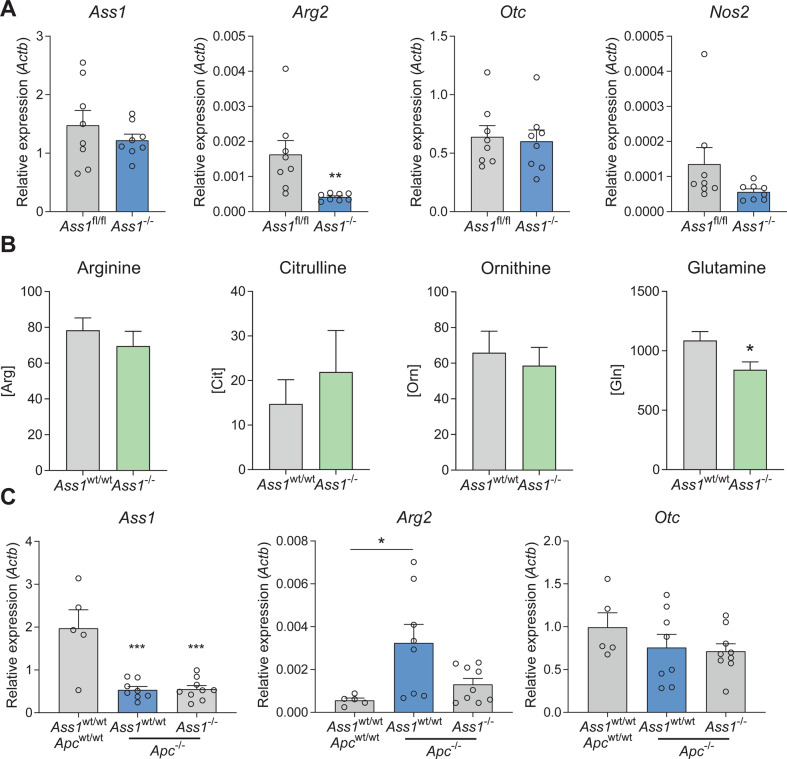
Plasma glutamine levels and expression of urea-cycle enzymes in the liver, might compensate for intestinal loss of *Ass1*. **A** qRT-PCRs for indicated genes in liver tissue from VillinCre^ERT2^ *Ass1*^wt/wt^ and VillinCre^ERT2^ *Ass1*^−/−^ mice 96 h after 14 Gy irradiation. *Actb* was used as a reference gene. ^**^p < 0.01 by Student’s *t*-test. **B** Plasma levels of indicated amino acids measured by HPLC from VillinCre^ERT2^ *Ass1*^wt/wt^ and VillinCre^ERT2^ *Ass1*^−/−^ mice 96 h after 14 Gy irradiation. ^*^p < 0.05 by Student’s *t*-test. **C** qRT-PCRs for indicated genes in liver tissue from VillinCre^ERT2^ *Ass1*^wt/wt^ *Apc*^wt/wt^, VillinCre^ERT2^ *Ass1*^wt/wt^ *Apc*^−/−^, and VillinCre^ERT2^ *Ass1*^−/−^ *Apc*^−/−^ mice, 5 days after induction. *Actb* was used as a reference gene. ^*^p < 0.05 by one-way ANOVA with Tukey’s multiple comparisons test.

*Ass1*-deficient epithelial cells are unable to synthesize arginine; so in the situation of an arginine-free diet these cells must receive arginine from another source. It was shown previously that altered amino acid metabolism in the liver can compensate for loss of *Ass1* during development [31]. Therefore, we measured gene expression in livers from the whole-body irradiation experiment (Fig. 4). Lack of intestinal epithelial *Ass1* in this model led to a significant decrease in hepatic *Arg2* expression (Fig. 8A), which would lead to decreased breakdown of arginine, to elevate systemic arginine levels in these mice. Plasma arginine levels were equal between wild-type and intestinal epithelial *Ass1* knockout mice. Other amino acid levels were also unaltered, except for glutamine, which was decreased in the *Ass1* knockout mice (Fig. 8B).

In VillinCre^ERT2^ *Ass1*^fl/fl^ *Apc*^fl/fl^ mice, *Ass1* expression in the liver was significantly reduced upon loss of *Apc* in the intestinal epithelium. However, the expression level of *Ass1* in the liver was not affected by the expression of *Ass1* in the epithelium, as liver *Ass1* expression was similar between *Apc*^*−/−*^*Ass1*^*wt/wt*^ and *Apc*^*−/−*^*Ass1*^*−/−*^ mice (Fig. 8C). In contrast, *Arg2* expression in the liver was clearly increased upon *Apc* loss, whereas this increase was reduced upon combined loss of *Apc* and *Ass1* (Fig. 8C). These data indicate that the increased synthesis of arginine in the intestinal epithelium in the context of regeneration or *Apc* knockout leads to systemic reduction of glutamine and hepatic alterations in amino acid metabolism, which can serve as a potential compensation mechanism.

## Discussion

It was previously suggested that *Ass1* contributes to intestinal regeneration and tumorigenesis, but our data from multiple in vitro and in vivo experiments do not support this hypothesis. Complete functional ablation of *Ass1* from cells of epithelial origin had no effect on tumorigenesis in a mouse model that is representative with the mutational spectrum of 80% of sporadic human colorectal cancers. Also, we found no role for epithelial *Ass1* during regeneration in a model of irradiation-induced epithelial damage and repair. *Ass1* expression is increased specifically by deletion of *Apc* and the urea-cycle flux is arrested. The correlation we find between *Apc* and *Ass1* is most likely mediated through translocation of beta catenin to the nucleus with subsequent activation of the Wnt target gene *cMyc*, which has been shown to bind to the promoter of *Ass1* and increase *Ass1* expression [42]. Other transcription factors that can indirectly be influenced by activation of the Wnt signaling pathway, such as HIF1alpha and ATF4, are likely to also influence *Ass1* expression [43, 44].

The loss of *Ass1* in the context of an *Apc* mutation does seem to lead to intrinsic compensation by amino acid transporters and extrinsic compensation by distant organs like the liver. As we show, arginine transporters are upregulated in *Ass1-*deficient organoids allowing more arginine uptake from the medium. At the same time, serum glutamine levels are increased and hepatocytes alter their arginine metabolism, by which the amino acid demand in the intestinal tissue can be met. This is similar to what was already reported during embryogenesis, although there are major differences in the response. In our models, breakdown of arginine by arginase is inhibited in the liver, whereas in embryogenesis, hepatic expression of arginase is increased [31]. These differences may be explained by the fact that arginine is required for the developing embryo, whereas the adult body is not in need of the excess of arginine that is produced upon a loss of *Apc*. Besides, newborn mice are not fed arginine before the suckling-to-weaning transition has been completed, as the mother’s milk does not contain any.

In the study of Miyamoto et al., in which *Ass1* heterozygosity led to aggravated damage after irradiation [27], the heterozygosity was not cell-type specific. In our experiments, the homozygous loss of *Ass1* was intestinal epithelium specific, with only *Villin*-expressing cells being affected. The effect of reduced synthesis of arginine in all cells apparently renders the body incapable of compensating for the loss, but this is not the case with our specific knockout. Although hepatic *Ass1* did not seem to contribute to the compensation for intestinal loss in our study, *Arg2* did. Thus, it may well be that the sum effect of body-wide *Ass1* heterozygosity in the study of Miyamoto et al. overloaded the compensatory mechanisms. In particular, non-epithelial intestinal cells, such as fibroblasts or immune cells, may play a role in this compensation, since they are in close vicinity to the epithelial cells and seem to express ASS1 in our experiments (Figs. 4C and 5H), but this remains to be elucidated.

It is likely that the high expression of *Ass1* in intestinal epithelial cells during regeneration serves the purpose of optimizing cellular growth and proliferation. However, multiple compensatory mechanisms, either cell-autonomous or systemic, are available to rescue local loss-of-function as is the case with many fundamental biological processes. Besides, as was previously shown, loss of *Ass1* may lead to an increase in cell division by facilitating pyrimidine synthesis via CAD (carbamoyl-phosphate synthase 2, aspartate transcarbamylase, and dihydroorotase complex) activation, through increased aspartate availability [19]. In addition, the strong inhibition of arginase in the hyperproliferative intestinal epithelial cells may have a stronger effect on the arginine availability than the increased *Ass1* expression [45].

A recent report has shown that some colorectal cancer cell lines have low ASS1 and OTC expression, which renders these cell lines arginine auxotrophic (dependant on extracellular arginine), similar to our *Apc*-*Ass1* knockout model [46]. This study shows that in a xenotransplantation model, arginine deprivation leads to inhibition of proliferation of the ASS1-low colorectal cancer cells. This is in contrast to our findings in vivo where loss of *Ass1* does not affect proliferation in an *Apc*-mutated background. In xenotransplantation models, cancer cells are injected subcutaneously, which might explain the differences with our model. Native stromal cells in the intestine might compensate for *Ass1* loss in the epithelium, whilst subcutaneous tissue (mostly adipocytes and fibroblasts) might not provide compensation. Also, colorectal cancer cell lines generally have acquired a large amount of mutations, which can influence metabolic needs and thereby lead to a different response from our more selectively mutated *Apc* model.

With regard to their arginine metabolism, hyperproliferative intestinal epithelial cells are reprogrammed in a similar way during the processes of embryonic development, regeneration after damage, and tumorigenesis. However, our results demonstrate that ASS1 is not essential for this reprogramming, since the functions related to ASS1 can be efficiently compensated for. An important consequence of this finding is that our data suggest that epithelial ASS1 may not be a potential therapeutic target for colorectal cancer treatment.

## Supplementary information

Supplementary figure legends.

Supplemental Figure 1.

Supplemental Figure 2.

Supplemental Figure 3.

Supplemental Figure 4.

## Data Availability

Data and materials are available through the accession number GSE179777 and the corresponding author on request.
