# Supplementary material for: Epithelial argininosuccinate synthetase is dispensable for intestinal regeneration and tumorigenesis
Source: Cell Death Dis. 2021 Oct 1;12(10):897. doi: 10.1038/s41419-021-04173-x (PMC8486827; doi:10.1038/s41419-021-04173-x)
Supplement: Supplementary file 1 — Supplementary figure legends. [file 41419_2021_4173_MOESM1_ESM.docx]

**Supplemental figure 1:**

**A:** Schematic overview of the urea cycle and associated processes. Processes as known from the literature, some reactions might not apply for intestinal epithelial cells. **B:** western blot for Arginase 2 in wildtype (WT; from C57BL/6 mice), *Apc*^fl/fl^, *Apc*^-/-^ organoids.

**Supplemental figure 2**:

**A:** *ASS1* expression in 20 colorectal cancer cell lines from **figure 2A**, separated by mutational status of indicated genes **B:** *ASS1* mRNA expression in 20 colorectal cancer cell lines from **figure 2A**, separated by CIMP (CpG island methylator phenotype) and MSI (microsatellite instability) status. **C:** pie-charts of staining intensity score distribution of **figure 2F**.

**Supplemental figure 3:**

**A:** Schematic overview of labeling in LC-MS experiments with labeled citrulline. **B:** Schematic overview of labeling in LC-MS experiments with labeled aspartic acid. **C:** relative labeling of fumarate in wildtype (WT) and *Apc*^-/-^ organoids incubated with labeled aspartic acid. **D:** Griess test for NO production in WT and *Apc*^-/-^ organoids. RAW 264.7 cells incubated with LPS were used as a positive control.

**Supplemental figure 4:**

**A:** qRT-PCR for indicated stem cell marker genes and Wnt-target gene *Axin2* in *Ass1* and *Apc*-deficient organoids **B:** clonogenic assay of *Ass1*^wt/wt^*Apc*^-/-^ and *Ass1*^-/-^*Apc*^-/-^ organoids, by seeding single cells in matrigel **C:** qRT-PCR for indicated arginine transporter genes in *Ass1* and *Apc*-deficient organoids. *Actb* and *Ppia* were used as reference genes.
