# Supplementary figures and images for: Epithelial argininosuccinate synthetase is dispensable for intestinal regeneration and tumorigenesis
Source: Cell Death Dis. 2021 Oct 1;12(10):897. doi: 10.1038/s41419-021-04173-x (PMC8486827; doi:10.1038/s41419-021-04173-x)

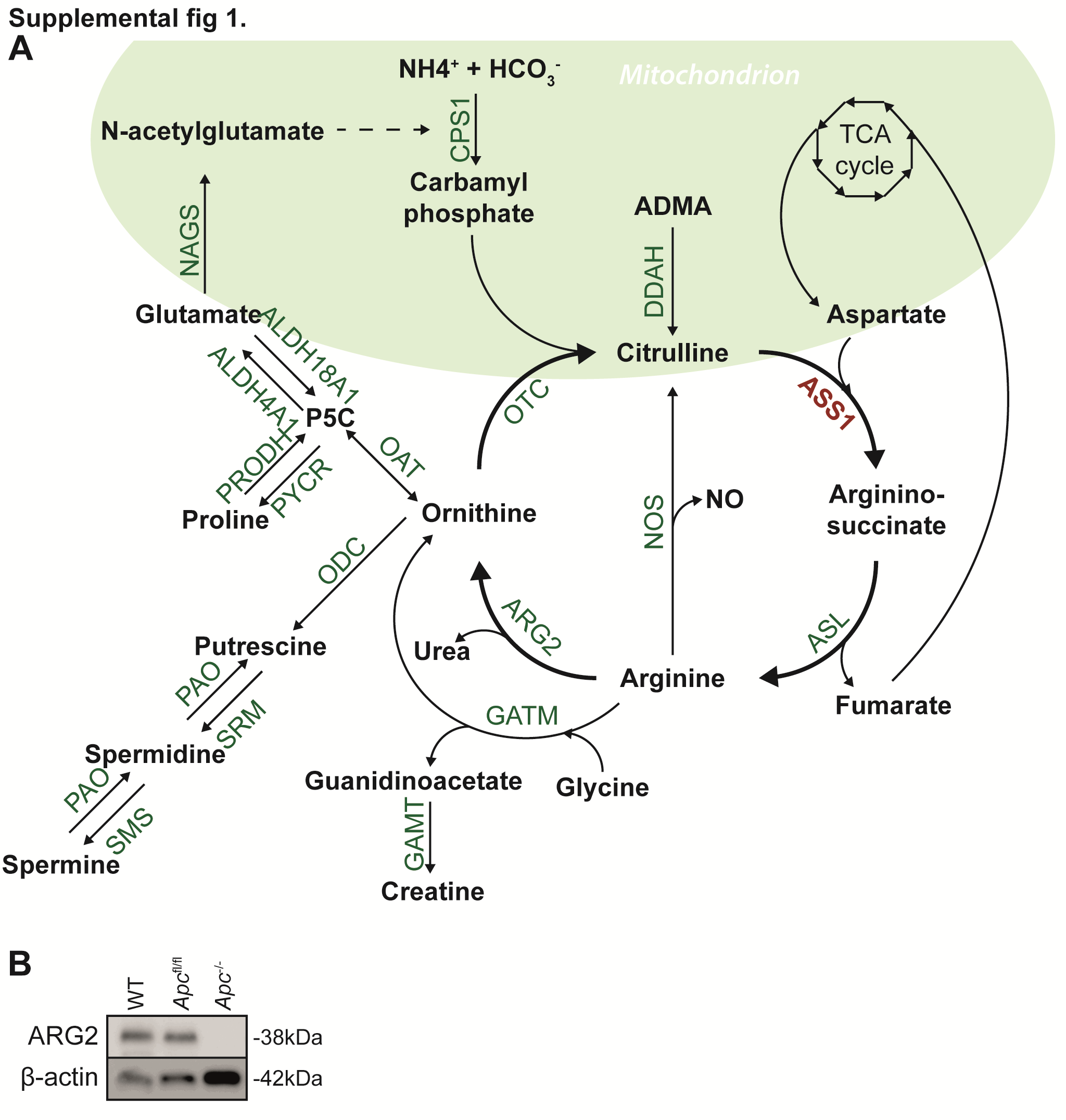

Supplement: Supplementary file 2 — Supplemental Figure 1. [file 41419_2021_4173_MOESM2_ESM.tif]

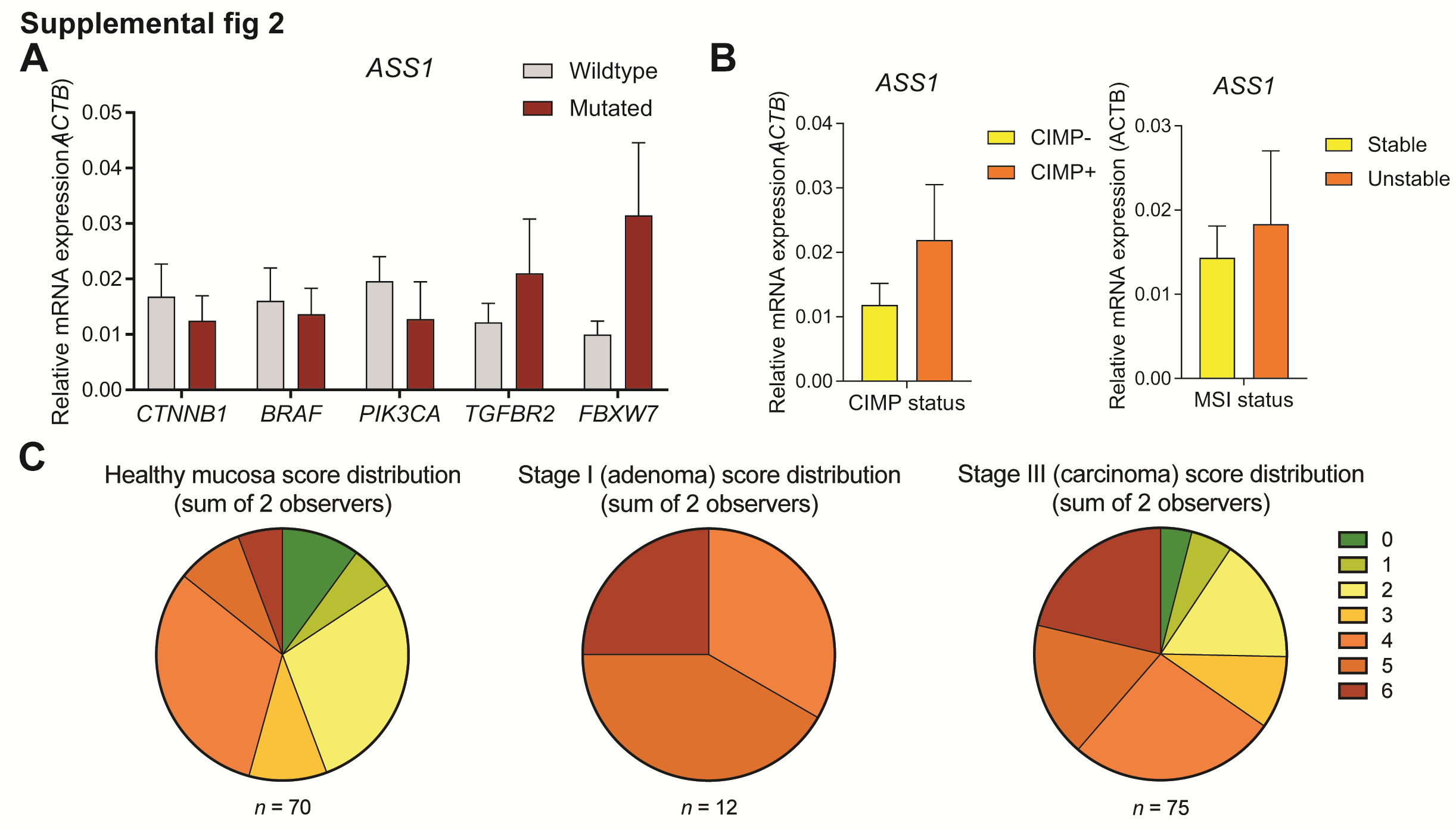

Supplement: Supplementary file 3 — Supplemental Figure 2. [file 41419_2021_4173_MOESM3_ESM.tif]

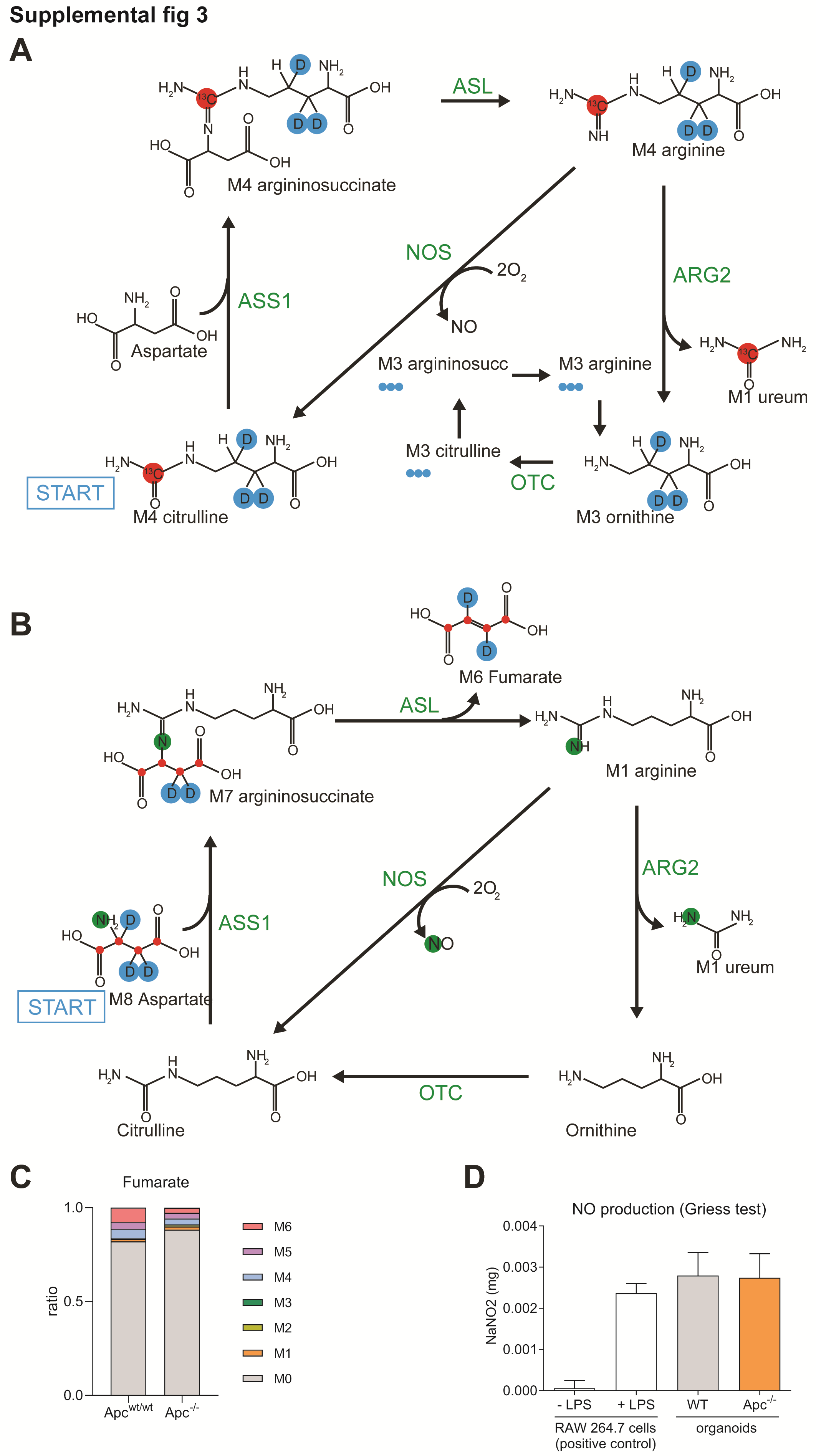

Supplement: Supplementary file 4 — Supplemental Figure 3. [file 41419_2021_4173_MOESM4_ESM.tif]

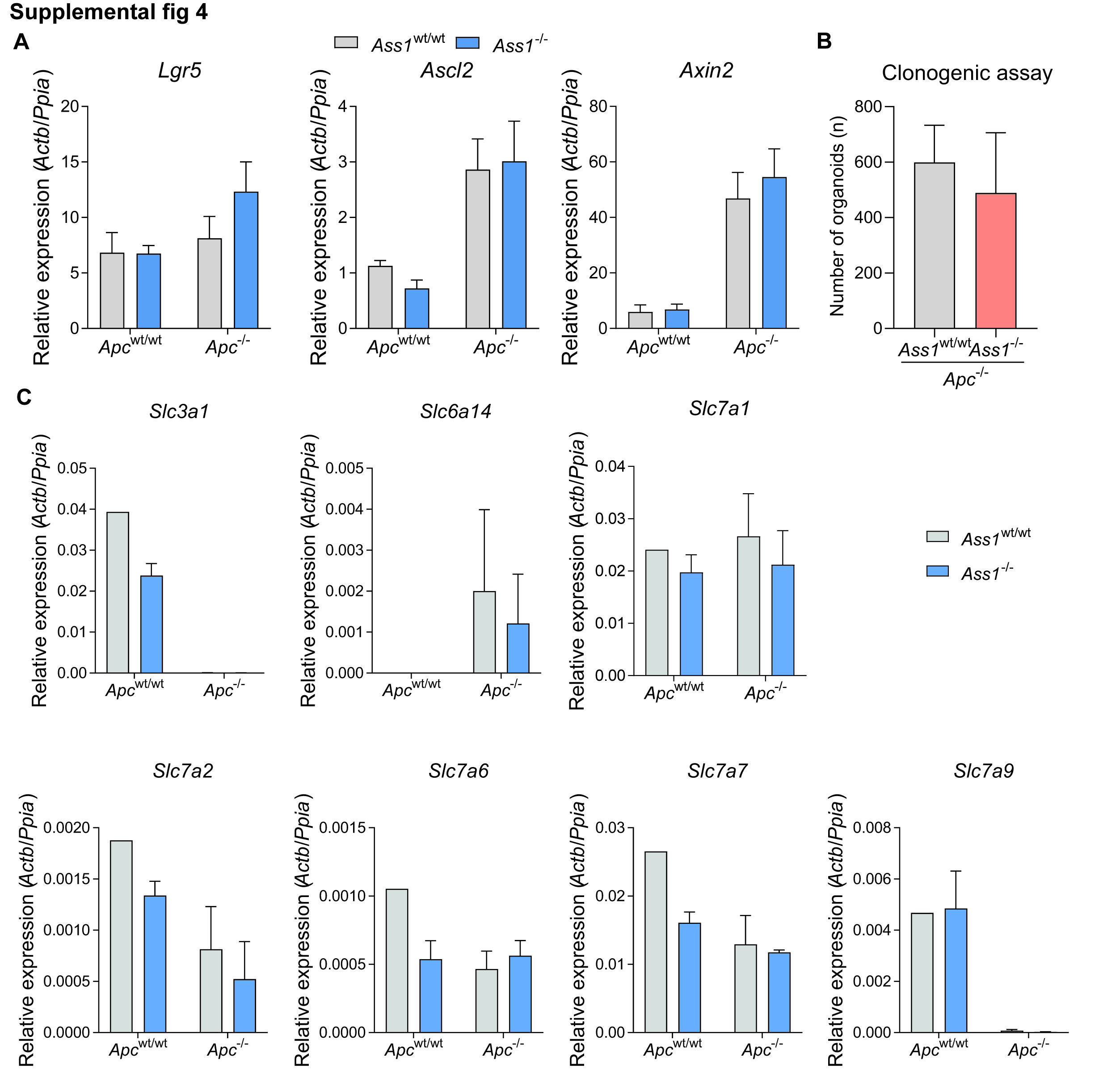

Supplement: Supplementary file 5 — Supplemental Figure 4. [file 41419_2021_4173_MOESM5_ESM.tif]
